# Minimal Access Tricuspid Valve Surgery

**DOI:** 10.3390/jcdd10030118

**Published:** 2023-03-13

**Authors:** Jean-Alexandre Sauvé, Yung-Szu Wu, Ravi Ghatanatti, Joseph Zacharias

**Affiliations:** Department of Cardiothoracic Surgery, Lancashire Cardiac Centre, Whinney Heys Road, Blackpool FY3 8NR, UK

**Keywords:** minimal access surgery, tricuspid valve regurgitation, endoscopic and robotic cardiac surgery

## Abstract

Tricuspid valve diseases are a heterogeneous group of pathologies that typically have poor prognoses when treated medically and are associated with significant morbidity and mortality with traditional surgical techniques. Minimal access tricuspid valve surgery may mitigate some of the surgical risks associated with the standard sternotomy approach by limiting pain, reducing blood loss, lowering the risk of wound infections, and shortening hospital stays. In certain patient populations, this may allow for a prompt intervention that could limit the pathologic effects of these diseases. Herein, we review the literature on minimal access tricuspid valve surgery focusing on perioperative planning, technique, and outcomes of minimal access endoscopic and robotic surgery for isolated tricuspid valve disease.

## 1. Introduction

Tricuspid valve disease is a heterogeneous group of pathologies that lead to the impairment of the valve’s normal function, which is to ensure the unidirectional flow of blood from the right atrium to the right ventricle. Functional abnormalities (either regurgitation or stenosis) resulting from disease are classified as primary and secondary. Primary causes affect the native tricuspid valve annulus or leaflets and include congenital (Ebstein’s), myxomatous degeneration, rheumatic heart disease, infective endocarditis, carcinoid syndrome, iatrogenic valve damage (device leads, endomyocardial biopsies), toxic aetiologies, tumours, and blunt trauma. In Europe, over 90% of tricuspid disease is secondary regurgitation due to pressure and/or volume overload mediated RV dilatation or due to an enlarged atrium/tricuspid annulus due to chronic atrial fibrillation [1]. The prevalence of significant moderate or severe tricuspid regurgitation (TR) increases with age; maybe as high as 0.55% of the general population and has a poor prognosis when left untreated [2,3]. Management of tricuspid valve disease includes medical treatment of the underlying pathology as well as interventional strategies. European guidelines recommend surgery for severe symptomatic primary tricuspid regurgitation, liberally during left-sided surgery in patients with secondary tricuspid regurgitation and significant tricuspid stenosis, as well as in patients with isolated symptomatic secondary disease appropriate for surgery and those with no/mild symptoms but with RV dilatation and severe tricuspid regurgitation [4,5]. A limiting factor of early intervention in isolated tricuspid valve disease is the significant morbidity and mortality associated with a standard sternotomy approach in these older patient populations [6,7]. Additionally, patients presenting late with advanced degrees of RV dilation/dysfunction and/or fixed pulmonary hypertension are unlikely to benefit from the surgery [8]. Minimal access tricuspid valve surgery (MATVS; direct vision, endoscopic and robotic) mitigates some of this risk which could allow for earlier and more appropriate timing of the intervention to promote reverse RV remodeling, avoid organ failure, and improve functional status [9,10].

## 2. Preoperative Planning

A patient with isolated tricuspid valve disease will initially be assessed using transthoracic echocardiography by his primary physician or cardiologist and once thresholds and guidelines are met will be referred to a surgical or multidisciplinary team to assess the feasibility of an attempt to repair or replace the valve. A new risk score for in-hospital mortality prediction after isolated tricuspid surgery can also be of use to inform the patient and physicians and guide the clinical decision-making process [7]. This score, called the TRI-SCORE, is derived from 466 patients in French centres during a 10-year period and uses a twelve-point scoring system based on eight easy-to-ascertain risk factors to establish a predicted in-hospital mortality rate which ranges from 1% (0 points) to 65% (≥9 points). Once the patient has been informed of the operative plan and has given appropriate consent, further preoperative planning to assess the suitability of the patient for a minimal access approach may proceed.

Patients will typically undergo a routine preoperative workup including a history and physical, a twelve-lead electrocardiogram, a chest X-ray, routine laboratory work, a transthoracic echocardiography, and a coronary angiogram. Due to selective lung ventilation during the procedure, pulmonary function tests are also commonly requested especially when the patient has a long-standing history of smoking or significant pulmonary disease. Depending on the clinician’s experience and expertise, an ECG gated contrast thoracoabdominal CT scan with follow-through to the femoral arteries may be requested for evaluation of the thoracic anatomy, cardiac chamber sizes and positioning, ascending aorta size, as well as vascular anatomy [11]. This evaluation is sometimes omitted by certain groups [12]. The use of ultrasound scanning is an alternative in experienced centres with vascular ultrasound expertise.

Commonly, surgeons will also plan their cardiopulmonary bypass strategies at this point with particular attention to cannulation, clamping, and cardioplegia delivery. It is, however, not uncommon for these plans to be modified/adapted to the clinical context at the time of the operation. In MATVS, venous cannulation is almost always peripheral either percutaneous or via cut-down. Arterial cannulation is also commonly peripheral with some groups opting for a central approach [12]. In the case of peripheral cannulation, the common femoral artery was the most common site (either percutaneously or via cut-down) followed by the axillary artery. A femoral artery diameter of 7 mm or more was considered suitable for a cannula starting at 21 Fr [13]. Clamping strategies are either direct using a Chitwood style external clamp or peripheral via an intra-aortic occlusion device (IntraClude device Edwards through an EndoReturn cannula). As per Edwards’ recommendation, an ascending aortic diameter of 40 mm or more is unfavourable for IntraClude utilization. Myocardial protection has been shown to be comparable between techniques in certain series [14]. Cardioplegia delivery is often antegrade with direct aortic cannulation or via the central lumen of the IntraClude device. A continuous retrograde delivery strategy has been utilized in some centres (ProPlege, Edwards) [15]. Most of the time, MATVS is performed on an arrested hypothermic heart although, when there is a contraindication to cross-clamping or use of the endovascular device, it may be performed on a beating/fibrillating heart [16].

## 3. Intraoperative Procedure

### 3.1. Anesthetic Consideration

General anaesthesia considerations vary widely between centres. MATVS are nearly always elective cases, although some urgent infective endocarditis cases have been reported in the literature [17]. Patients are positioned in the supine position with an inflatable bag or roll placed either transversally at the level of the 4th intercostal space or vertically at the level of the spine. They will typically have the standard anaesthesia monitoring of the respiratory and circulatory system including pulse oximetry, inspired oxygen analyser, capnography, measurement of pulmonary mechanics, non-invasive and invasive blood pressure monitoring (bilateral radial arterial lines often when IntraClude is used to monitor the migration of the balloon), electrocardiogram leads, defibrillation pads, temperature probe(s), peripheral and urinary catheter, quantitative neuromuscular monitoring, and transoesophageal echocardiography (TOE). Cerebral (and/or peripheral) oximetry monitoring and depth of anaesthesia/EEG surveillance are also commonly used. Centres describe a variety of ventilation strategies including intermittent ventilation of a single-lumen endotracheal (ET) tube or lung isolation via either double-lumen ET tubes or single-lumen ET tubes with bronchial blockers [18,19]. Once the patient is under general anaesthesia, central venous lines are placed and, occasionally, Swan-Ganz catheters may be used. Other invasive lines and monitoring may be used depending on the patient’s clinical condition and risk factors.

Often, the superior vena cava (SVC) cannula is inserted by the anaesthetist at this stage via the internal jugular vein and properly positioned with the aid of TOE. In redo cases, an arterial embolectomy catheter can be used and inserted through a custom-assembled side arm to occlude SVC before the right atrial opening [13].

### 3.2. Surgical Technique

**Figure 1:**
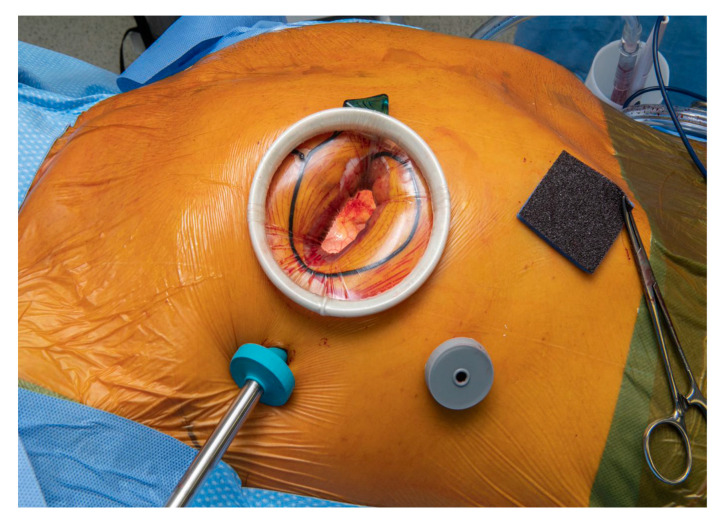
An example of the typical setup for MATVS at the Lancashire cardiac centre. A periareolar skin incision is made with a mini-thoracotomy at the fourth intercostal space with a camera port at the same level (blue trocar). An additional trocar (grey) is placed to facilitate the installation of a cardiotomy succion and/or pericardial stay sutures.

Once the patient is positioned, skin disinfected, and draped with sterile sheets, the surgery commences. The skin incision used in isolated MATVS varies between centres but has trended to smaller incisions with the introduction of video assistance. A periareolar skin incision is typically the most cosmetic, delivering patient-satisfying results [20,21,22]. It can be extended laterally when needed to accommodate for a prosthesis when the valve cannot be repaired or when extensive lysis of adhesions is required. Blunt dissection and muscle splitting of the pectoral muscle allow for entry into the thoracic cavity; sometimes, the muscle is simply divided with diathermy for better haemostasis. Soft tissue retractors are recommended, and a thoracic spreader may be used in certain cases when using direct vision. Additional ports are also installed via 5–10 mm stab incisions; typically, a camera port will be installed in the anterior axillary line at either the 3rd or 4th intercostal space (ICS). A working port at the level of the 4th or 5th ICS (we prefer the 4th ICS; see Figure 1) and an atrial retractor incision may also be made medial to the working port in the 4th ICS. A further 5 mm port is used to introduce a cardiotomy suction device just above the diaphragm in the 6th space in the mid-axillary line. We also use this port to insufflate carbon dioxide. Left atrial retractors may be used, although a novel dedicated right atrial retractor has recently been developed [23]. Cameras used in endoscopic surgery may be 2D or 3D and of varying resolution. Advantages of 3D cameras are enhanced anatomical details and depth perception as well as easier skill acquisition and transfer [24]. Robotic MATVS shares many similarities with the endoscopic techniques. Port configurations are similar: the working port which is typically placed in the third intercostal space (ICS) at the anterior axillary line, the left arm port which is placed in the second ICS (halfway between anterior axillary and midclavicular line), the right arm port in the fifth ICS below the anterior axillary line, and the atrial retractor which is placed in the fourth ICS, 2 cm medial to midclavicular line [25].

As described above, several vascular cannulation strategies are possible and there is great variability between centres. Most commonly, an SVC cannula is inserted by the anaesthetist, and inferior vena cava cannulation (IVC) is performed either percutaneously or via cutdown of the common femoral vein by the surgeon. The IVC cannula is advanced to the junction of the IVC and right atrium. Other strategies of venous cannulation with good outcomes include direct bicaval cannulation using the standard venous cannulas or single multi-stage femoral cannulation [26,27]. An atrial venous shunt may be used once the atriotomy has been performed to provide for adequate drainage of the SVC via the single IVC cannula in certain cases [28]. Snaring of the venae cava can be achieved directly through standard snares or with large bulldog vascular clamps in non-redo operations [13]. In redo cases, arterial embolectomy catheters or vascular occlusion balloons have also been used either through contralateral insertion sites and advancement past the venous cannulas’ tips or through a side port of the cannulas themselves (as explained above) [13,29,30]. Others have shown that performing tricuspid surgery without caval occlusion is safe due to the air being captured by the active drainage system [31]. This requires very good communication with the perfusion team to avoid air lock of the venous line in case of excessive vacuum assist.

**Figure 2:**
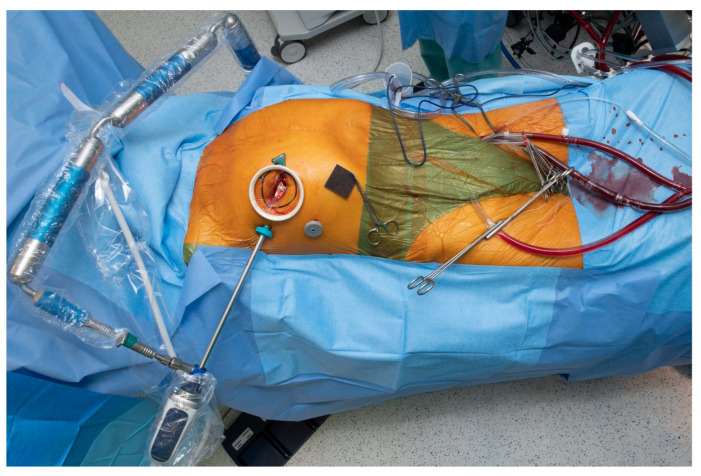
Full cannulation setup. Of note, this case was done on a beating heart with a single venous cannula and no additional shunting or occlusion devices. Furthermore, a ‘Y’ arterial connection is routinely constructed to facilitate additional arterial cannulation if need be (doubly clamped arterial connection at the bottom of the image). The camera holder is a pneumatically operated arm which can be seen at the top of the image.

Arterial cannulation in the setting of MATVS is typically femoral but may occasionally be axillary, central, and even, although rarely, via the carotid artery [32]. It may be percutaneous when aortic cross-clamping is done centrally or when no clamping is envisioned. It is more likely by cutdown if the IntraClude device is used given the required larger size of the inflow cannula [33]. Different cardioplegic strategies are possible when arrest is desired with most centres opting for single-dose cardioplegia when available. Other centres avoid cross-clamping by performing beating heart surgery or on a hypothermic fibrillating heart, especially in redo cases (see Figure 2) [16,34].

Techniques for repair and replacement in MATVS are identical to those in open surgery [5]. Knot-tying may be achieved with automatic devices (such as CorKnot, which have several potential intraoperative advantages described elsewhere) or with knot-pushing devices [35]. Given the steep learning curve of MATVS, several learning tools have been suggested. Notably, a ‘suture map’ has been developed to guide surgeons in the placement of their sutures during annuloplasty repair. A reverse backhand position is suggested for the initial three sutures along the anterior leaflet and the course of the right coronary artery; a reversed forehand position for the next two sutures along the commissure between the anterior and posterior leaflets; a forehand position for the next three sutures along the mid portion of the posterior leaflet to the mid portion of the septal leaflet [36].

The most common method for pacing after an isolated MATVS is to place standard epicardial pacing wires directly on the surface of the right ventricle [13]. In redo cases, where dissection of adhesions is to be avoided, pacing can be achieved through the tricuspid valve using a transvenous pacing catheter inserted preoperatively and repositioned after the repair or replacement [37]. In some cases, the standard epicardial pacing wires may be placed directly into the endocardial surface of the right ventricle and passed through the tricuspid valve and right atriotomy suture line [13]. After weaning from CPB, loose closure of the pericardium with a small drain in the pericardium and another drain in the right pleura is common. In redo cases, a single drain in the right pleura without closing the pericardium may be warranted [13].

## 4. Postoperative Course

Postoperative care of MATVS is akin to that of sternotomy patients. Fluid replacement in patients with long-term dependence on high-dose diuretic therapy can be challenging. Close attention needs to be given to the right ventricular end-diastolic volumes as overloading the right ventricle in the post-tricuspid valve repair setting can have fatal consequences. Wound monitoring at peripheral cannulation is important, and special attention should be given to the potential development of vascular complications in the initial phase with femoral arterial cannulation (more at risk with percutaneous cannulation) [38,39]. Vascular assessment of peripheral pulses with a doppler and pencil probe may be considered in case of concern. Additionally, given the thoracic approach of MATVS, care must be given to ensure appropriate lung re-expansion after surgery, which can be assessed by chest X-ray. Patients are slightly more at risk of air leaks than their counterparts undergoing open surgery and of pleural effusion/haemothorax, particularly in the initial stages. Some centres advocate for the quick removal of drains, within 24–48 h post-surgery, while others prefer to leave the pericardial drain in place for several days to prevent late pericardial effusion [40,41].

## 5. Outcomes of MATVS

Minimal access cardiac surgery (MACS) has been shown in multiple case series and propensity-matched analysis to have several advantages when compared to traditional open sternotomy: smaller incisions resulting in less pain, better cosmesis, faster recovery, reduced blood loss, shorter hospital stays, and lowered risk of wound infection [42,43,44]. When focusing specifically on MATVS, risk/benefit assessments are more difficult to evaluate given that there is a distinct lack of randomized controlled trials comparing the outcomes of MATVS to those undergoing surgery through standard sternotomy when contrasted to minimal access approaches to mitral and aortic valve disease [45]. In 2020, our group summarized our outcomes which included isolated MATVS but also MATVS as a concomitant procedure and in redo settings [10]. Morbidity including cerebrovascular accident (CVA) varied from 0% and 4%, renal complications around 8–13% (which was lower than the 24–35% rate in sternotomy patients), and mortality estimated between 4.1 and 17% in these heterogeneous groups [12,13,40,46,47,48].

MACS has traditionally been reported to have a higher risk of CVAs as compared to standard sternotomy, which was believed to be due to the use of retrograde cardiopulmonary bypass, leading some centres to shift towards using antegrade cardiopulmonary bypass methods [49]. Recent studies have found, however, that the risk of CVA is similar to the use of retrograde perfusion in selected patients [50]. MATVS in reported series has been shown to have a 0–4% risk of CVA, which is akin to the rates in meta-analyses of open surgery comparing repair vs. replacement (stroke risk 1.5% vs. 0.9%) [51,52].

Patients undergoing isolated tricuspid valve surgery may be at a higher risk of renal complications due to their comorbidities and often delayed referral for surgery. Studies have shown higher rates of renal failure and acute kidney injury (AKI) in patients undergoing redo tricuspid valve surgery, with some studies reporting incidence as high as 30–35% with sternotomy [40,53]. However, studies comparing MATVS to sternotomy have generally reported a lower risk of AKI and a new onset dialysis requirement in the MATVS group. These studies have reported new onset renal replacement therapy ranging from 5.6% to 13% in the MATVS groups as compared to 12.4–15.6% (repair vs. replacement) in the traditional literature [40,51,54]. In high-risk patients undergoing MATVS, the risk of acute renal failure requiring renal replacement therapy is reported to be around 7.8% [46].

One of the causes of the increasing diagnosis of tricuspid valve regurgitation is the increased prevalence of atrial fibrillation (AF) in the general population and the increasing use of device leads [55,56]. Many patients undergoing tricuspid valve surgery, therefore, have underlying rhythm problems and the surgery itself is at risk of causing such issues given the anatomical proximity of the tricuspid valve to the conduction system [36]. Tricuspid valve surgery, therefore, carries a high risk of post-operative arrhythmias and device implantation; with variable rates in the defined MATVS case series.

Tricuspid valve surgery via sternotomy has been shown to have high mortality rates, reaching up to 50% in some cases [2]. On the other hand, MATVS seems to have lower mortality rates when compared to the sternotomy approach. Studies have reported a 30-day mortality rate of 5% for both sternotomy and MATVS in redo settings [40]. In contrast, Färber et al. showed a mortality rate of 8% in primary MATVS and 7% in the redo MATVS group compared to 27% in the redo sternotomy group [47]. Abdelbar et al. showed a 30-day mortality rate of 4.1% in MITS both in primary and redo settings [13]. Additionally, Chen and colleagues have reported a significant reduction in mortality after shifting from median sternotomy to MITS, from 23.3% to 4.6% [48]. While associated with adverse perioperative outcomes, in a series of 79 patients undergoing endoscopic repeat isolated tricuspid valve surgery (RITS) after left-sided valve replacement (LSVR) endoscopic access was acceptable as an alternative to sternotomy [57]. Further retrospective analyses of RITS after LSVR have revealed no difference in early mortality and long-term survival between patients undergoing an endoscopic approach vs. sternotomy, although patients undergoing endoscopic approaches had shorter ICU stays and fewer reoperations [58,59]. MATVS as an isolated procedure in a redo setting was also shown to be a safe procedure with no reported mortality in 47 patients [60].

### Potential Additional Benefits of MATVS

As reported previously, MACS has been shown to have several potential benefits when compared to open surgery, and this likely applies to MATVS as well. There are, therefore, likely benefits regarding operative times, blood loss, pain management, and overall cost-benefit advantages. Operative times for MACS have traditionally been longer than for open surgery [61]. As surgical techniques have progressed and technological advances have been made, operative time for MACS cases has consequently diminished. In addition, some studies have reported shorter bypass times for MATVS than median sternotomy, particularly in redo surgery (78 ± 31 min vs. 91 ± 23 min) [40]. Despite this, the use of an endovascular occlusion clamp in some cases can increase bypass times. In any case, operative times for MATVS did not seem to impact mortality. MACS has been shown to have a lower incidence of blood loss compared to standard sternotomy due to its smaller incision and lack of bone division [45]. Studies have also shown that MATVS results in less bleeding during the first 24 h post-surgery, with lower mean total drainage [40]. Re-exploration for bleeding is also lower in redo MATVS as compared to sternotomy [47]. This is reflected in the lower blood transfusion rates after redo MATVS. Additionally, Chen and colleagues reported no blood transfusion at all in their redo MATVS group [48]. As most patients referred for tricuspid valve surgery are likely to have some liver congestion, smaller incisions are more likely to reduce the bleeding risks. There are limited reports specifically on pain scores related to isolated MATVS; however, there is a wealth of evidence detailing to benefits of MACS on pain reduction and faster return to normal activity as compared to standard sternotomy [62,63,64]. It is believed that the pain reduction is due to preserving sternal integrity, resulting in better thorax stability [65]. While not specific to MATVS, quality-of-life studies have reported that up to 94% of patients reported no or minimal pain after their MACS procedure, and 43% reported having returned to work within three weeks [66]. In more recent years, regional and local anaesthesia techniques have been utilized to reduce post-operative pain even further, with studies reporting a reduction in post-operative narcotics usage and improved pain scores [67,68].

## 6. Conclusions

In summary, isolated MATVS is a safe and reproducible procedure that can be used to effectively address multiple causes of tricuspid valve disease and notably deal with secondary causes of tricuspid regurgitation, which are becoming more prevalent as the population ages. It offers several potential advantages when compared to open surgery including a lower risk of renal complications, fewer blood transfusions, lesser device implantations, and lower mortality. It also has other benefits such as improved cosmesis, less pain, decreased wound infections, and earlier mobilization. Further studies will now be needed to identify the optimal timing for surgery for isolated functional TR and to truly establish whether an intervention such as surgery can confer survival benefits when compared to medical therapy alone; with current evidence suggesting it may without reaching statistical significance [4,69]. Although current evidence supports the timing range on a variety of factors (including patient characteristics, disease aetiology, and anatomical considerations), and that earlier interventions before significant right ventricular dysfunction, congestive heart failure, and pulmonary hypertension seem to offer better results, additional studies including randomized controlled trials would be of use [5]. Finally, such an approach will eventually need to be compared to transcatheter techniques which are rapidly evolving and for which patient outcome studies are now being conducted [70,71].
